# MeConcord: a new metric to quantitatively characterize DNA methylation heterogeneity across reads and CpG sites

**DOI:** 10.1093/bioinformatics/btac248

**Published:** 2022-06-27

**Authors:** Xianglin Zhang, Xiaowo Wang

**Affiliations:** Ministry of Education Key Laboratory of Bioinformatics, Center for Synthetic and Systems Biology, Bioinformatics Division, Beijing National Research Center for Information Science and Technology, Department of Automation, Tsinghua University, Beijing 100084, China; Ministry of Education Key Laboratory of Bioinformatics, Center for Synthetic and Systems Biology, Bioinformatics Division, Beijing National Research Center for Information Science and Technology, Department of Automation, Tsinghua University, Beijing 100084, China

## Abstract

**Motivation:**

Intermediately methylated regions occupy a significant fraction of the human genome and are closely associated with epigenetic regulations or cell-type deconvolution of bulk data. However, these regions show distinct methylation patterns, corresponding to different biological mechanisms. Although there have been some metrics developed for investigating these regions, the high noise sensitivity limits the utility for distinguishing distinct methylation patterns.

**Results:**

We proposed a method named MeConcord to measure local methylation concordance across reads and CpG sites, respectively. MeConcord showed the most stable performance in distinguishing distinct methylation patterns (‘identical’, ‘uniform’ and ‘disordered’) compared with other metrics. Applying MeConcord to the whole genome data across 25 cell lines or primary cells or tissues, we found that distinct methylation patterns were associated with different genomic characteristics, such as CTCF binding or imprinted genes. Further, we showed the differences of CpG island hypermethylation patterns between senescence and tumorigenesis by using MeConcord. MeConcord is a powerful method to study local read-level methylation patterns for both the whole genome and specific regions of interest.

**Availability and implementation:**

MeConcord is available at https://github.com/WangLabTHU/MeConcord.

**Supplementary information:**

Supplementary data are available at *Bioinformatics* online.

## 1 Introduction

**Fig. 1.**
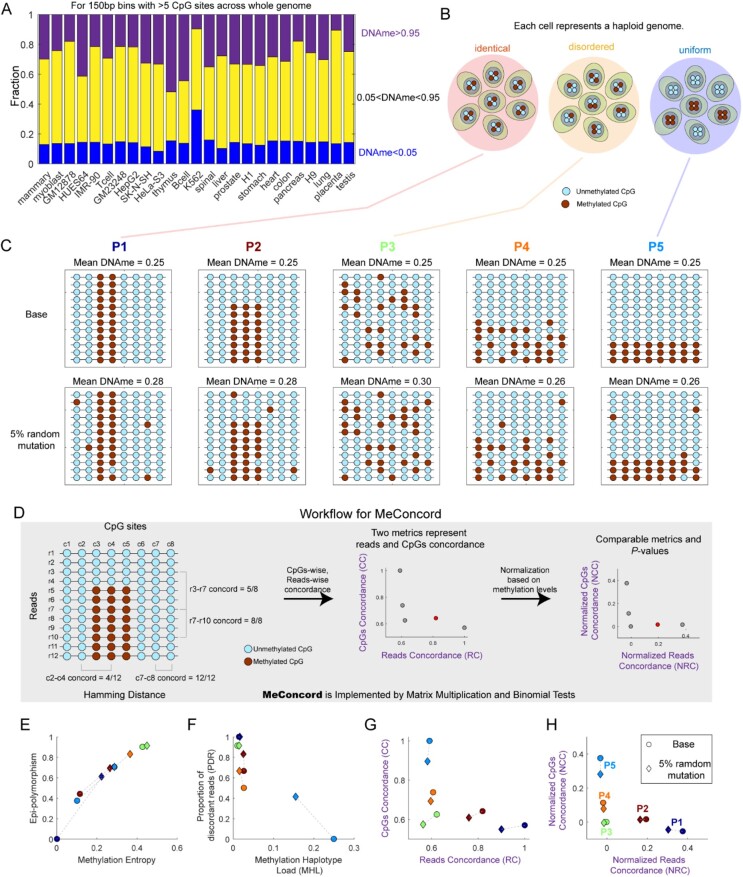
The motivation of this study and the workflow of MeConcord. (**A**) The fractions of 150-bp bins (>5 CpG sites) with different DNA methylation levels. Intermediately methylated bins are defined as bins with methylation levels >0.05 and <0.95 and occupy a large fraction (0.33–0.76, median = 0.57) across the whole genome. (**B**) A cartoon represents three different methylation patterns for intermediately methylated regions. Each cell represents a haploid genome. (**C**) Five different methylation patterns for intermediately methylated regions (methylation levels were around 0.25 for each pattern) with 8 CpG sites and 12 reads. Top: methylation patterns without methylation mutations. Bottom: methylation patterns with 5% random methylation mutations to mimic real experiment data. Patterns 1, 3 and 5 correspond to the cartoon shown in panel B. (**D**) The workflow of Meconcord. Methylation concordance was calculated across reads (reads concordance) and CpG sites (CpGs concordance) by applying Hamming distance to pairwise comparisons. Reads concordance and CpGs concordance are biased by mean methylation levels, so expected reads concordance and expected CpGs concordance were calculated based on the number of methylated and unmethylated CpGs across all pairwise comparisons. Normalized reads concordance and normalized CpGs concordance were defined by the differences between raw and expected scores. *P*-values were calculated by using Binomial tests. (**E–H**) The scatter plots of metrics measuring regional methylation heterogeneity for 10 methylation patterns including both the base and mutated patterns. E and F show four metrics used in previous studies, exhibiting poor performance in distinguishing different methylation patterns. However, G and H show metrics used in this study, exhibiting good performance in distinguishing different methylation patterns, especially for P1, P3 and P5, and in coping with noise

DNA methylation is one of the most pervasive and well-studied epigenetic modifications in mammalian genomes. In human genome, intermediately methylated regions (0.05 < DNA methylation < 0.95) occupied 33–76% for 150-bp bins harboring more than 5 CpG sites (Fig. 1A;  Supplementary Fig. S1 for more stringent cutoffs). There mainly are three methylation patterns (‘identical’, ‘disordered’, ‘uniform’, Fig. 1B and C) in these regions (Derrien *et al.*, 2021; Landan *et al.*, 2012). These patterns usually imply different biological mechanisms and can arise from a mix of different cell types or cell states, genomic imprinting, DNA methylation erosion and dynamic competition between DNMT and TET. Intermediately methylated regions are informative regions, as they either act as potential features used for cell-type deconvolution in bulk data (Li *et al.*, 2018; Zheng *et al.*, 2014) or underlie the diversity and heterogeneity of cell states which contribute to gene expression regulation (Derrien *et al.*, 2021; Landau *et al.*, 2014). Quantitatively characterizing and distinguishing different DNA methylation patterns of these regions are markedly valuable for studying the underlying regulation mechanisms and developing new biomarkers.

Because single bisulfite sequencing reads often cover multiple CpG sites at one time, read-level methylation analysis could uncover the methylation patterns in these intermediately methylated regions. However, it requires a suitable metric to efficiently describe and distinguish different methylation patterns. At present, methylation entropy (Jenkinson *et al.*, 2017; Xie *et al.*, 2011), epi-polymorphism (Landan *et al.*, 2012), proportion of discordant reads (PDR) (Landau *et al.*, 2014) and fraction of discordant reads pairs (FDRP) (Scherer *et al.*, 2020) were proposed to describe within-sample methylation heterogeneity of reads. Although these metrics have been successfully used in some specific conditions, they always harbor two disadvantages. One is the neglect of the concordance between adjacent CpG sites. Methylation entropy, epi-polymorphism and FDRP focused on the methylation differences between reads but did not consider the association between local CpG sites. The other is high sensitivity to methylation noise. DNA methylation is highly dynamic and a snapshot of DNA methylation for a sample is noisy. The noise could come from both technical noise and biological noise. For example, the former could be caused by incomplete bisulfite conversion (Olova *et al.*, 2018); the latter could result from a competition between DNMT and TET during DNA replication and transcription (Charlton *et al.*, 2018; Ginno *et al.*, 2020; Kangaspeska *et al.*, 2008; Métivier *et al.*, 2008). Methylation entropy, epi-polymorphism and PDR showed poor performance in coping with the simulated noisy data (Fig. 1C, E and F). Besides, methylation haplotype load (MHL) is a metric used to amplify the differences between fully consecutively methylated and interspersedly methylated patterns (Guo *et al.*, 2017) and has limited application scenarios. All these drawbacks limit the investigation of intermediately methylated regions.

In this study, we leveraged Hamming distance to define two metrics to characterize DNA methylation concordance at read and CpG levels, respectively. Two metrics and their corresponding *P*-values calculated by Binomial tests clearly distinguished three methylation patterns in intermediately methylated regions and showed good performance in coping with noisy data. Using MeConcord, we found distinct methylation patterns were associated with different genomic characteristics, such as CTCF binding and genomic imprinting. Further, we identified the differences of CpG island’s hypermethylation patterns between senescence and tumorigenesis by using MeConcord. MeConcord is a powerful method to study local methylation patterns for both the whole genome and specific regions of interest.

## 2 Materials and methods

### 2.1 Overview

To quantitatively measure DNA methylation heterogeneity in intermediately methylated regions, we introduced concordance scores at two dimensions—concordance between reads and concordance between CpG sites. For example, Figure 1D showed the fractions of concordant CpG pairs for Reads 3 and 7 (5/8), Reads 7 and 10 (8/8), where numerator values were concordant CpG pairs (with the same methylation states at the same CpG sites for two reads), while denominator values were the number of all valid pairs (with coverages at the same CpG sites for two reads). Similarly, for the other dimension, the fractions of concordant CpG pairs for CpG sites 2 and 4 (4/12), CpG sites 7 and 8 (12/12) were shown in Figure 1D.

Iteratively counting concordant CpG pairs and all valid CpG pairs across possible pairwise comparisons of reads, we could obtain reads concordance (RC) scores by dividing all concordant CpG pairs by all valid CpG pairs. Similarly, we could also get CpGs concordance (CC, short for CpG sites concordance) scores (The middle of Fig. 1D). Matrix multiplication was used to speed up the calculation (see following methods and more details with an example shown in Supplementary Fig. S2).

Reads concordance and CpGs concordance are not comparable, because they are biased by mean methylation levels (Supplementary Fig. S3). So, we calculated expected concordance scores under random conditions for the given methylation data to normalize reads concordance and CpGs concordance, and calculated corresponding *P*-values by Binomial tests (the right of Fig. 1D; more calculation details in Supplementary Fig. S2) to show the significance of concordance.

### 2.2 Calculating reads concordance and CpGs concordance

This section is the implementation of reads concordance and CpGs concordance with matrix multiplication. First, to be compatible with missing data in methylation matrix (reads × CpG sites), we defined methylated matrix M, with element scores 1 indicating methylated CpGs and 0 indicating missing data or unmethylated CpGs; unmethylated matrix N, with element scores 1 indicating unmethylated CpGs and 0 indicating missing data or methylated CpGs. The coverage matrix T is the summarization of M and N, with element score 1 indicating coverages and 0 indicating missing data. Matrix M, N and T have the same sizes, with the number of rows r representing the number of reads and the number of columns c representing the number of CpG sites on the specific region, which was 150-bp bins in this study. 150 bp was chosen due to its compatibility with the length of next-generation sequencing reads.

Then, reads concordance (*RC*) was calculated as

(1) $$mr=\sum_{\text{all}\;\text{elements}}\left(MM^{T}⊙(U_{r\times r}-I_{r})\right)$$

 

(2) $$nr=\sum_{\text{all}\;\text{elements}}\left(NN^{T}⊙(U_{r\times r}-I_{r})\right)$$

 

(3) $$tr=\sum_{\text{all}\;\text{elements}}\left(TT^{T}⊙(U_{r\times r}-I_{r})\right)$$

 

(4) RC=(mr+nr)/tr

where mr, nr, tr represent the numbers of concordantly methylated CpG pairs, concordantly unmethylated CpG pairs, all valid CpG pairs across all possible pairwise comparisons of reads, respectively. Besides, $U_{r\times r}$ represents an all-ones matrix with size r×r, where r is the number of reads, which is equal to the row numbers of matrix M, N and T. $I_{r}$ represents an identity matrix with all-ones in the main diagonal and all-zeros for other elements. ⊙ represents dot product.

Similarly, CpGs concordance (CC) was calculated by counting all concordant CpG pairs across all pairwise comparisons of CpG sites (Fig. 1D). CC was implemented as

(5) $$mc=\sum_{\text{all}\;\text{elements}}\left(M^{T}M⊙(U_{c\times c}-I_{c})\right)$$

 

(6) $$nc=\sum_{\text{all}\;\text{elements}}\left(N^{T}N⊙(U_{c\times c}-I_{c})\right)$$

 

(7) $$tc=\sum_{\text{all}\;\text{elements}}\left(T^{T}T⊙(U_{c\times c}-I_{c})\right)$$

 

(8) CC=(mc+nc)/tc

where mc, nc, tc represents the numbers of concordantly methylated CpG pairs, concordantly unmethylated CpG pairs, all valid CpG pairs across all possible pairwise comparisons of CpG sites, respectively. Besides, $U_{c\times c}$ represents an all-ones matrix with size c×c, where c is the number of CpGs, which is equal to the column numbers of matrix M, N and T. $I_{c}$ represents an identity matrix with size c×c.

### 2.3 Calculating normalized concordance metrics and *P*-values

We noticed that reads concordance and CpGs concordance were biased by DNA methylation levels. More close to 0.5 the methylation level is, lower values two metrics are (Supplementary Fig. S3). So we computed expected reads concordance and expected CpGs concordance with the methylation matrix and further normalized two metrics by subtracting the raw concordance by the expected concordance.

Normalized reads concordance (NRC) was calculated as

(9) $$mp_{r}=\sum_{\text{all}\;\text{elements}}\left(TM^{T}⊙(U_{r\times r}-I_{r})\right)$$

 

(10) $$np_{r}=\sum_{\text{all}\;\text{elements}}\left(TN^{T}⊙(U_{r\times r}-I_{r})\right)$$

 

(11) $$pr=mp_{r}/(mp_{r}+np_{r})$$

 

(12) $$er=pr^{2}+{\left(1-pr\right)}^{2}$$

 

(13) NRC=RC−er

where $mp_{r}$, $np_{r}$ represent the numbers of methylated CpGs, unmethylated CpGs in all possible CpG pairs across all pairwise comparisons of reads, respectively. pr represents the expected methylation level according to the composition of CpG pairs, er represents the expected reads concordance given the expected methylation level pr.

To represent the significance of concordance over expectations under random conditions, we leveraged Binomial tests to calculate *P*-values to represent the significance of concordance for the methylation matrix. *P*-values of reads concordance ($P_{r}$) was calculated as

(14) $$P_{r}=\{\begin{array}{c} \sum_{i=0}^{mr+nr}\left(\begin{array}{c} tr\\ i \end{array}\right)er^{i}{\left(1-er\right)}^{tr-i},\;\;if\;mr+nr<tr\times er\\ \sum_{i=mr+nr}^{tr}\left(\begin{array}{c} tr\\ i \end{array}\right)er^{i}{\left(1-er\right)}^{tr-i},\;\;if\;mr+nr\geq tr\times er \end{array}$$

where mr+nr is the observed counts; tr is all possible counts; er is the expected reads concordance.

Similarly, normalized CpGs concordance (*NCC*) was calculated as

(15) $$mp_{c}=\sum_{\text{all}\;\text{elements}}\left(T^{T}M⊙(U_{c\times c}-I_{c})\right)$$

 

(16) $$np_{c}=\sum_{\text{all}\;\text{elements}}\left(T^{T}N⊙(U_{c\times c}-I_{c})\right)$$

 

(17) $$pc=mp_{c}/(mp_{c}+np_{c})$$

 

(18) $$ec=pc^{2}+{\left(1-pc\right)}^{2}$$

 

(19) NCC=CC−ec

where $mp_{c}$, $np_{c}$ represents the numbers of methylated CpGs, unmethylated CpGs shown in all possible CpG pairs across all pairwise comparisons of CpG sites, respectively. pc represents the expected methylation level according to the composition of CpG pairs, ec represents the expected reads concordance given the expected methylation level pc.

*P*-values of CpGs concordance ($P_{c}$) was calculated as

(20) $$P_{c}=\{\begin{array}{c} \sum_{i=0}^{mc+nc}\left(\begin{array}{c} tc\\ i \end{array}\right)ec^{i}{\left(1-ec\right)}^{tc-i},\;\;if\;\;mc+nc<tc\times ec\\ \sum_{i=mc+nc}^{tc}\left(\begin{array}{c} tc\\ i \end{array}\right)ec^{i}{\left(1-ec\right)}^{tc-i},\;\;if\;\;mc+nc\geq tc\times ec \end{array}$$

### 2.4 Data collection and processing

We collected whole genome bisulfite sequencing data of human samples from both ENCODE (Davis *et al.*, 2018) and previously published datasets (Cruickshanks *et al.*, 2013; Jenkinson *et al.*, 2017) under GEO accession GSE48580 and GSE86340. Bam files mapped to GRCh38 under ENCODE3 version with Bismark were directly used. Raw sequencing data from GEO was used followed by quality control, adapter sequences removal and mapping. We employed Cutadapt (Martin, 2011) to remove adapter sequences. Then Bismark (Krueger and Andrews, 2011) was used to map sequencing reads against GRCh38.

Bam files of transcription factors and histone modifications ChIP-seq, DNase-seq for H1 and K562 cell lines were downloaded from ENCODE and used for the enrichment. The feature enrichment was performed by deepTools (Ramírez *et al.*, 2016).

Human reference genome GRCh38 was used in this study. The genome was binned at 150-bp windows (bins) and bins with more than 5 CpG sites were used for MeConcord analyses.

### 2.5 MeConcord implementation and functions

MeConcord was implemented by Python and included three main functions.

1. Converting bam files from Bismark to reads-level methylation recording files. Each single-end read or paired-end read was converted to one methylation recording considering the overlapping of two ends of paired-end reads. This part was compatible with both sam files (or sam.gz files) and bam files with even a mix of single-end and paired-end reads. This part required reads ID sorted files.
2. Calculating reads concordance, CpGs concordance, normalized reads concordance, normalized CpGs concordance and corresponding *P*-values from methylation recording files for any given genomic regions. Because this part was implemented by matrix multiplication, the coverage of reads could be more than 1000×, much higher than FDRP, which is compatible with less than 40× (Scherer *et al.*, 2020). This enabled MeConcord coping with target sequencing data with extremely high sequencing depths, such as RRBS.
3. Deriving methylation matrix (reads × CpG sites) from methylation recording files for any given genomic regions. The methylation matrices could be used for visualization by plotting lollipop plots (PDF format) with the script embedded in MeConcord.

### 2.6 Identifying differentially methylated regions on CpG islands

To well study the differences of CpG island hypermethylation patterns between cellular senescence, aging and tumorigenesis, at first we binned CpG islands into 150-bp bins and calculated mean methylation levels for each cell state or tissue. Next, we chose bins whose mean methylation levels in control samples (proliferating cells for cellular senescence, T cells from young individuals for aging, normal liver and lung tissues for tumorigenesis) <0.20 and standard deviations of control samples <0.05. Finally, differentially methylated regions for each case sample were independently identified with the threshold that methylation levels of the case sample minus mean methylation levels of controls >0.1. Methylation patterns were analyzed for these differentially methylated bins of CpG islands.

## 3 Results

### 3.1 MeConcord showed good performance in distinguishing different methylation patterns

First, we borrowed the naming of three canonical methylation patterns in intermediately methylated regions from a previous study (Derrien *et al.*, 2021), where the ‘identical’ methylation pattern represents a pattern with high consistency between reads but low concordance across adjacent CpG sites; the ‘disordered’ methylation pattern represents a pattern with low concordance both between reads and CpG sites; the ‘uniform’ methylation pattern represents a pattern with highly concordant CpG sites within single reads but large differences between some reads (Fig. 1B and C).

To examine the performance of MeConcord in distinguishing different methylation patterns, we simulated 5 methylation matrices with similar methylation levels, corresponding to different methylation patterns (Fig. 1C). Bottom of Figure 1C showed the simulated noisy methylation matrices to mimic real data. Both raw concordance scores (reads concordance and CpGs concordance) and normalized concordance scores [normalized reads concordance (NRC) and normalized CpGs concordance (NCC)] showed good performance in distinguishing five different methylation patterns (Fig. 1G and H). In contrast, previously used metrics showed poor performance in distinguishing these patterns, especially for the noisy matrices. Besides, methylation entropy, epi-polymorphism, PDR and MHL showed large differences between the base methylation matrices and the noisy methylation matrices, whereas our metrics, NRC and NCC, showed more similar scores. This suggested the better performance of our metrics in coping with noise. Our metrics also showed more stable performance than other metrics when applying them to shorter stretches of four CpG sites (Supplementary Fig. S4). Notably, Patterns 2 and 4 are not canonical patterns, therefore was excluded in the following analyses.

**Fig. 2.**
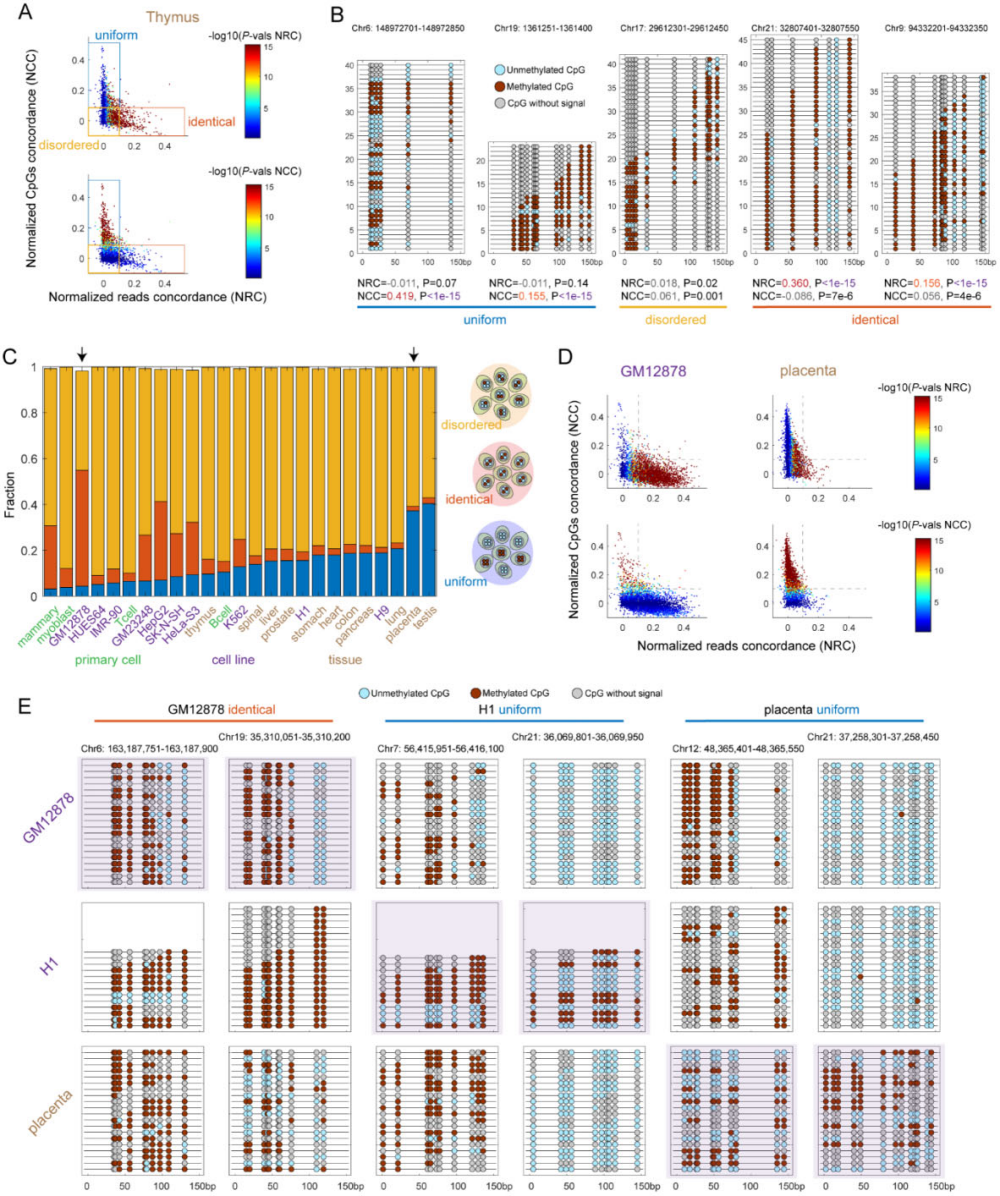
Distinct methylation patterns identified by MeConcord. (**A**) The distribution of normalized concordance scores and the classification of methylation patterns. Colors indicated the significances of concordance (*P*-values) at two different dimensions for top and bottom scatterplots, respectively. Each point represented a 150-bp bin with more than 5 CpG sites and more than 5 mapped reads. Shown points were subsampled at 1% from the whole gnome. (**B**) Lollipop plots of five examples for distinct methylation patterns. (**C**) The compositions of three distinct methylation patterns for 25 samples from ENCODE. These analyses were performed for intermediately methylated regions (0.05 < DNAme < 0.95). (**D**) The distributions of normalized concordance scores for GM12878 cell line and placenta tissue, which represented two extremes of the compositions. Top and bottom plots are colored by two types of *P*-values. (**E**) Lollipop plots for six genomic regions corresponding to GM12878-specific ‘identical’ patterns, H1-specific ‘uniform’ patterns and placenta-specific ‘uniform’ patterns, respectively

To examine MeConcord’s performance in real data, we performed the analysis on a thymus tissue sample from ENCODE (Supplementary Fig. S5, Fig. 2A). ‘Uniform’ regions exhibited a pattern that DNA methylation of CpGs on the same reads are highly consistent and there is a divergence among reads (left two examples of Fig. 2B), and were defined as bins showing high NCC (>0.1), low NRC (<0.1) and low *P*-values (<1 × 10^−10^) (Top square of Fig. 2A). Similarly, ‘disordered’ and ‘identical’ regions are defined as low NCC (<0.1), low NRC (<0.1) and high *P*-values (>1 × 10^−5^); low NCC (<0.1), high NRC (>0.1) and low *P*-values (<1 × 10^−10^), respectively (Fig. 2A and B). In contrast, other existing metrics could not efficiently distinguish different patterns in real data (Supplementary Fig. S6). Taken together, MeConcord was a reliable method for investigating methylation patterns in intermediately methylated regions.

### 3.2 Different proportions of distinct methylation patterns across samples

To investigate the different compositions of methylation patterns in different samples, we applied MeConcord to 25 samples from ENCODE, including 4 primary cells, 10 cell lines and 11 tissues. In general, ‘disordered’ bins occupied the highest fractions (43%–91%) (Fig. 2C). The proportions of ‘identical’ and ‘uniform’ bins showed large differences across different cell types/tissues (the proportions of ‘identical’ bins from 2% in placenta to 50% in GM12878; the proportions of ‘uniform’ bins from 3% in mammary epithelial cells to 40% in testis) (Fig. 2C and D). We found that tissues always showed higher proportions of ‘uniform’ bins than primary cells or cell lines, especially for placenta and testis. We speculated that multiple cell types within tissues contributed to a high fraction of ‘uniform’ bins. There were two exceptions; H1 and H9 cell lines showing high fraction of ‘uniform’ bins. However, This is in line with the previous finding that a high proportion of CpGs (14%) genome-wide showed allele-specific methylation in H1 and H9 (Chen *et al.*, 2011). More stringent cutoffs for intermediately methylated regions did not change these trends (Right part of Supplementary Fig. S1). Figure 2E showed some examples for GM12878-specific ‘identical’ bins, H1-specific ‘uniform’ bins and placenta-specific ‘uniform’ bins.

To well understand the biological relevance of these different methylation patterns in GM12878, H1 and placenta, we performed gene function enrichments. GREAT genomic enrichments (McLean *et al.*, 2010) of GM12878 ‘disordered’, ‘identical’ and ‘uniform’ bins showed different functions and phenotypes, and ‘uniform’ bins were enriched for genomic imprinting (Binomial *P*-values < 1 × 10^−30^) (Supplementary Fig. S7). Similarly, we found that H1 ‘uniform’ bins were enriched for embryonic morphogenesis (Binomial *P*-values < 1 × 10^−7^) and imprinting (Binomial *P*-values < 1 × 10^−40^) (Supplementary Fig. S8); placenta ‘uniform’ bins were enriched for embryonic placenta development (Binomial *P*-values < 1 × 10^−10^) and imprinting (Binomial *P*-values < 1 × 10^−30^). Interestingly, placenta ‘disordered’ bins were enriched for trophoblast (will develop into a large part of the placenta) morphology (Binomial *P*-values < 1 × 10^−10^) and abnormal placenta size (Binomial *P*-values < 1 × 10^−10^) (Supplementary Fig. S9). Taken together, different local methylation patterns are related to genomic regions with different biological functions.

### 3.3 Genomic features for three different methylation patterns

**Fig. 3.**
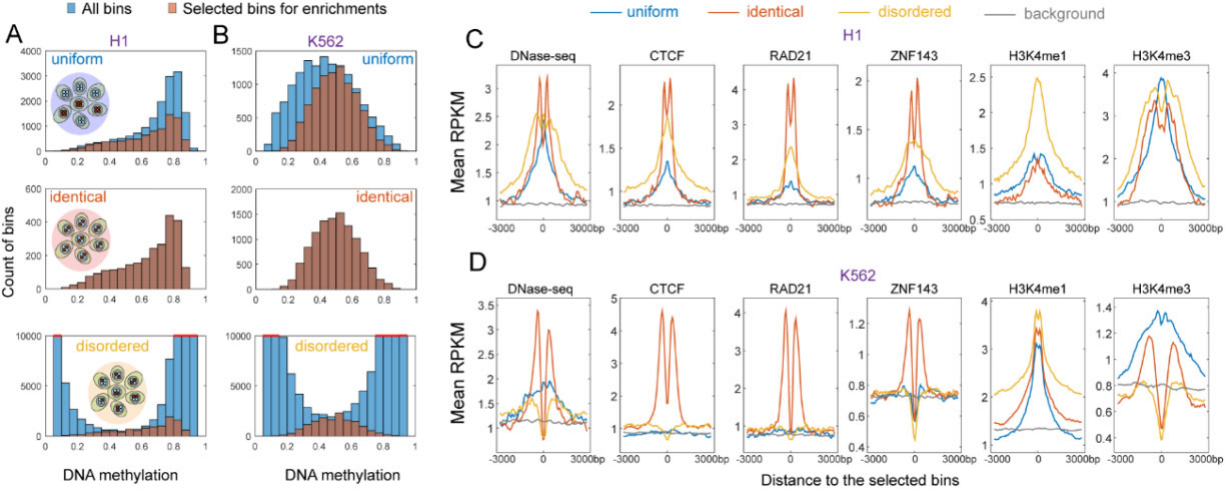
Different genomic features for three methylation patterns. (**A**) The distributions of DNA methylation levels for different methylation patterns in H1 cell line (All bins). We randomly selected bins of ‘uniform’ and ‘disordered’ in each methylation level interval to make their distributions be consistent with the distribution of ‘identical’ bins (Selected bins for enrichments). These selected bins were used for genomic feature enrichment analyses. This could largely avoid the effects of different DNA methylation levels on feature enrichments. (**B**) The distributions of DNA methylation levels for different methylation patterns in K562 cell line. (**C**) The enrichment of DNase-seq and ChIP-seq signal (CTCF, RAD21, ZNF143, H3K4me3, H3K4me1) around different methylation patterns for H1. The signal was shown upstream 3000 bp and downstream 3000 bp of these bins. We chose the 150 bp bins, which were 100 kbp upstream and downstream from the foreground bins (‘uniform’, ‘identical’, ‘disordered’), as the background regions. (**D**) The enrichment of DNase-seq and ChIP-seq signal around different methylation patterns for K562

To better understand the differences between three methylation patterns, we included H1 and K562 cell lines, which have comprehensive ChIP-seq data for various transcription factors and histone modifications in ENCODE, to investigate the relationship between these methylation patterns and these genetic and epigenetic characteristics. First, we examined the distributions of DNA methylation levels for three methylation patterns and found that ‘uniform’ and ‘identical’ bins showed similar methylation level distributions, whereas ‘disordered’ bins showed distinct methylation levels (Fig. 3A and B). To avoid the effect of different methylation levels of three methylation patterns on the following genomic feature enrichment analyses, we randomly selected genomic regions of ‘uniform’ and ‘disordered’ patterns in each methylation level interval to make sure that they showed similar distributions of methylation levels as ‘identical’ pattern (Selected bins of Fig. 3A and B).

The enrichments indicated that ‘identical’ bins were specifically associated with CTCF, RAD21 and ZNF143 bindings and placed around but not within these binding sites (Fig. 3C and D). It suggested that ‘identical’ patterns might be mediated by genome insulators or 3D genome organizers. ‘Disordered’ bins were depleted for H3K4me3, a mark of promoter, but enriched for H3K4me1, a mark of active and poised enhancer. In contrast, ‘uniform’ bins were enriched for both H3K4me3 and H3K4me1. These findings indicated that three different methylation patterns harbored distinct genomic characteristics.

### 3.4 Distinct CpG island hypermethylation patterns between senescence and tumorigenesis

To further examine the power of MeConcord to investigate methylation patterns, we leveraged datasets of cellular senescence, aging and tumorigenesis to study the differences of CpG island hypermethylation between senescence and tumorigenesis. Previous studies have shown that tumors and senescent cells harbors similar methylation alterations (Cruickshanks *et al.*, 2013; Zane *et al.*, 2014), but this finding is upsetting as it will be more difficult to specifically detect tumors from aged individuals in clinical care with DNA methylation markers. However, the differences between local methylation patterns might give some insights into this problem.

**Fig. 4.**
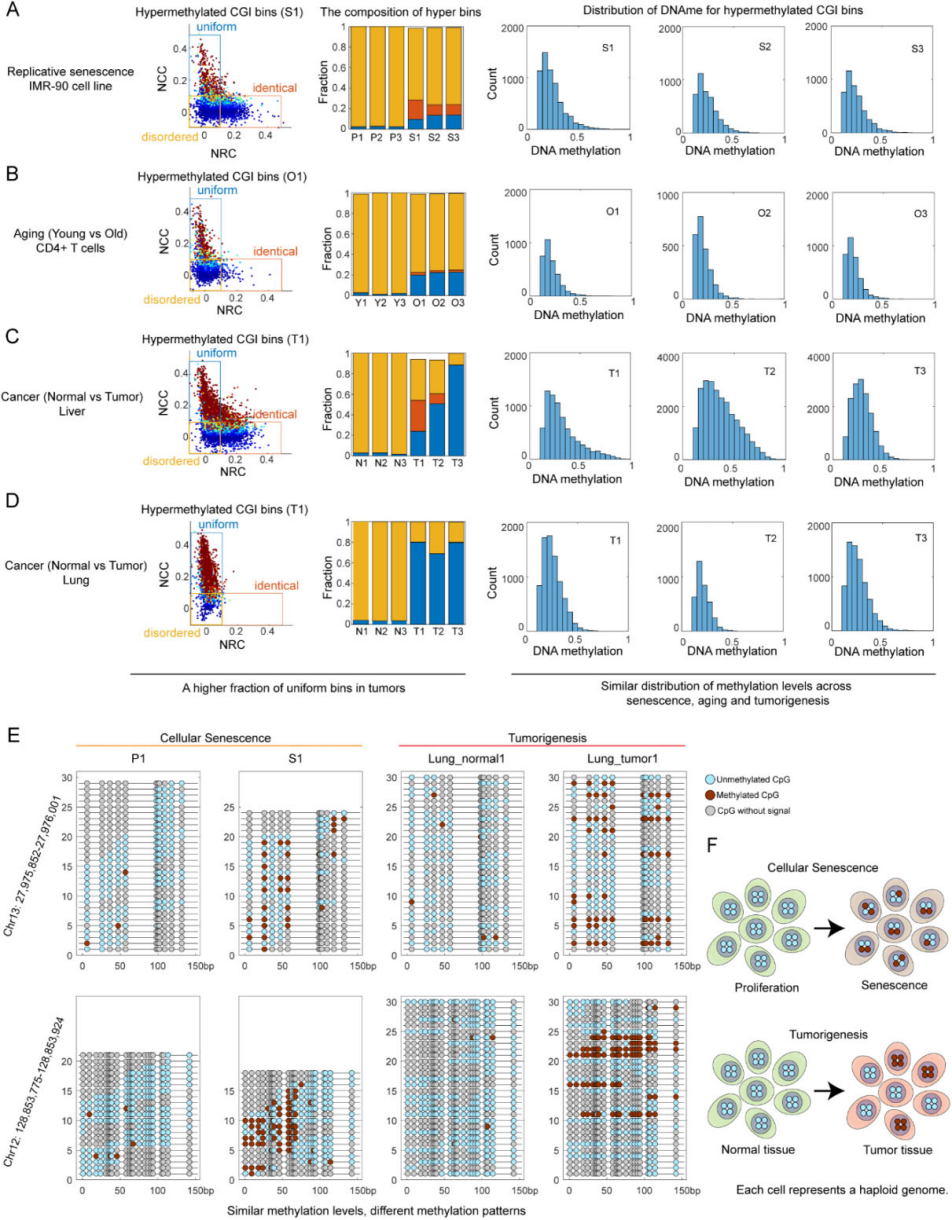
Distinct CpG island hypermethylation patterns in senescence and tumorigenesis. (**A–D**) The compositions of different methylation patterns and distributions of DNA methylation levels for hypermethylated CpG islands in cellular senescence (A), aging (B), liver cancer (C) and lung cancer (D). The scatterplots on the left showed the distributions of normalized concordance scores in one senescence sample, one aging sample or one tumor sample. Bar plots in the middle showed the compositions of three methylation patterns across all samples in each dataset. Tumor samples showed higher fraction of ‘uniform’ bins than cellular senescence samples or aging samples. Histograms on the right showed the distributions of DNA methylation of hypermethylated CpG islands for all case samples (senescence samples, aging samples, tumors). Except for liver tumor 2, all case samples showed similar distributions. (**E**) Lollipop plots of two examples for cellular senescence and tumorigenesis. The scores for MeConcord and existing metrics were shown in Supplementary Tables S1 and S2. For all metrics, NCC and its *P*-values of MeConcord showed the clearest differences between the senescence sample S1 and the lung cancer 1. One of the examples (Chr12:128 853 775–128 853 924) is located at the promoter of *GLT1D1*, which is associated with the occurrence of chronic obstructive pulmonary disease (Morrow *et al.*, 2016) and immunosuppression in B-cell lymphoma (Liu *et al.*, 2020). However, the relationship between different methylation patterns and biological mechanisms remains unclear. (**F**) A cartoon showing the differences of CpG island hypermethylation patterns between cellular senescence and tumorigenesis

We collected four datasets including cellular senescence dataset (3 proliferating biological replicates, 3 replicative senescence biological replicates), aging dataset (T cells from 3 young individuals and 3 old individuals), liver cancer dataset (3 normal liver tissues, 3 liver tumor tissues), lung cancer dataset (3 normal lung tissues, 3 lung tumor tissues). We focused on CpG island bins which showed hypermethylation during senescence or tumorigenesis and found that tumors showed higher fractions of ‘uniform’ bins than senescent cells (tumors, 0.24–0.88, mean = 0.65; senescence or aging, 0.10–0.23, mean = 0.17). In contrast, the distributions of DNA methylation levels for these hypermethylated CpG island bins were similar between senescence and tumorigenesis (Fig. 4A–D). It suggested that local methylation patterns identified by MeConcord were more suitable for distinguishing senescence and tumorigenesis than DNA methylation levels.

Figure 4E showed two examples of hypermethylated regions in both senescent cells and lung tumors. CpG islands of senescent cells tended to be methylated by ‘disordered’ or ‘identical’ patterns, whereas tumor tissues tended to be methylated by a ‘uniform’ pattern. For single reads, senescent cells tended to be methylated at some discrete CpG sites, while tumors tended to be methylated at nearly all consecutive CpG sites (Fig. 4E and F). Although ‘uniform’ patterns in tumor samples might be due to tumor purity, read-level methylation patterns revealed by MeConcord could help us to distinguish tumors and senescent cells.

## 4 Discussion

In this study, we introduced a new method, MeConcord, for analyzing local DNA methylation patterns at single-read level. This method leveraged Hamming distance, matrix multiplication and Binomial tests to overcome some limitations that present methods are facing. DNA methylation data was noisy due to the dynamics of DNA methylation mechanisms (Charlton *et al.*, 2018; Métivier *et al.*, 2008) and technical noise, which required metrics to have a good ability to cope with noisy methylation data. However, methylation entropy, epi-polymorphism and PDR either required very high coverages or focused on very local regions (4 CpG sites) to reduce the effect of noise. In contrast, MeConcord and quantitative fraction of discordant reads pairs (qFDRP) (Scherer *et al.*, 2020) leveraged Hamming distance to improve the stability in real datasets. Although one of our metrics, reads concordance, is equal to 1-qFDRP, qFDRP alone is impossible to distinguish three different methylation patterns. To our knowledge, there was no such a method distinguishing three distinct methylation patterns in intermediately methylated regions before MeConcord.

We leveraged matrix multiplication to calculate two concordance metrics, with an ability to include very-high-coverage datasets than qFDRP. This method can easily deal with >1000 reads for a single bin, while qFDRP had to subsample reads when the number exceeds 40 reads (Scherer *et al.*, 2020), which hampers its utility in deep sequenced datasets.

Present metrics including methylation entropy, epi-polymorphism and our raw metrics RC, CC were biased by DNA methylation levels (Landan *et al.*, 2012; Shao *et al.*, 2014) (Supplementary Fig. S3). This hampers us to set thresholds to distinguish different methylation patterns. However, we computed the expected concordance scores under random conditions for a given methylation matrix to normalize RC and CC, which is more efficient than permutations (Tsai *et al.*, 2012), and leveraged Binomial tests to assign a *P*-value for each score. This enabled us to easily distinguish different methylation patterns by setting an unbiased threshold.

By applying Meconcord to different samples and biological processes, we found that different methylation patterns identified by MeConcord harbored different genomic features and MeConcord could enable us to study the local read-level methylation patterns for both the whole genome and some specific regions. Currently, MeConcord can only process DNA methylation of CpG sites, and does not include non-CpG methylation frequently appeared in brains, stem cells and plant cells. However, MeConcord is a powerful method to study local read-level methylation patterns on CpG sites for intermediately methylated regions.

## Supplementary Material

btac248_Supplementary_Data
