# Supplementary material for: MeConcord: a new metric to quantitatively characterize DNA methylation heterogeneity across reads and CpG sites
Source: Bioinformatics. 2022 Jun 27;38(Suppl 1):i307–15. doi: 10.1093/bioinformatics/btac248 (PMC9235486; doi:10.1093/bioinformatics/btac248)
Supplement: btac248_Supplementary_Data [file btac248_supplementary_data.pdf]

# Supplementary Figures and Tables

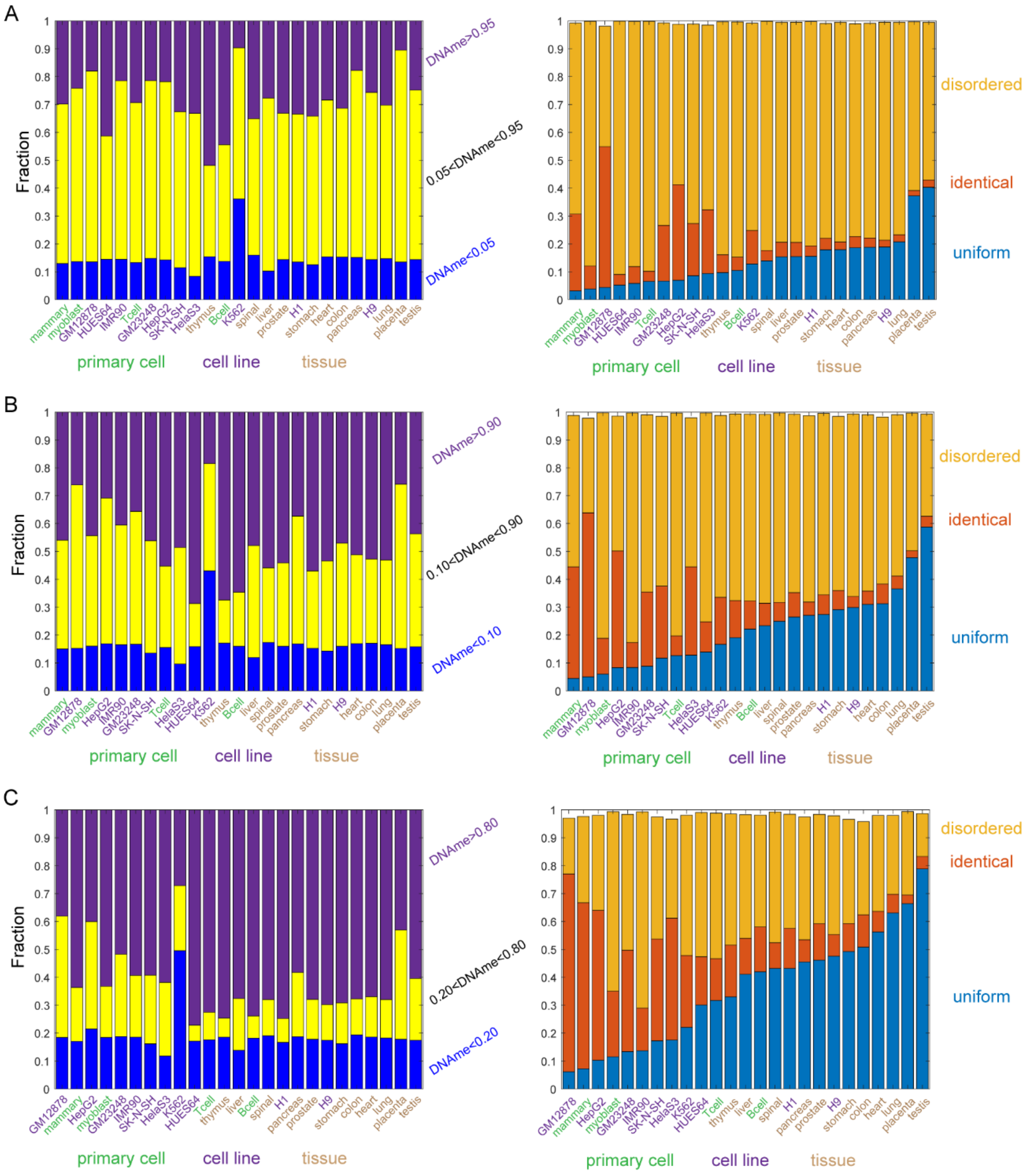


**Supplementary Fig. S1. The comparisons of different cutoffs for intermediately methylated regions.** (**A-C**) **Left**: The fractions of intermediately methylated regions for different cutoffs. (**A**) 33%–76% for the cutoff 0.05–0.95; (**B**) 15%–59% for the cutoff 0.10–0.90; (**C**) 6%–43% for the cutoff 0.20–0.80. **Right**: The fractions of different methylation patterns for intermediately methylated regions under different cutoffs. Different cutoffs have little effect on the ranking of different samples.

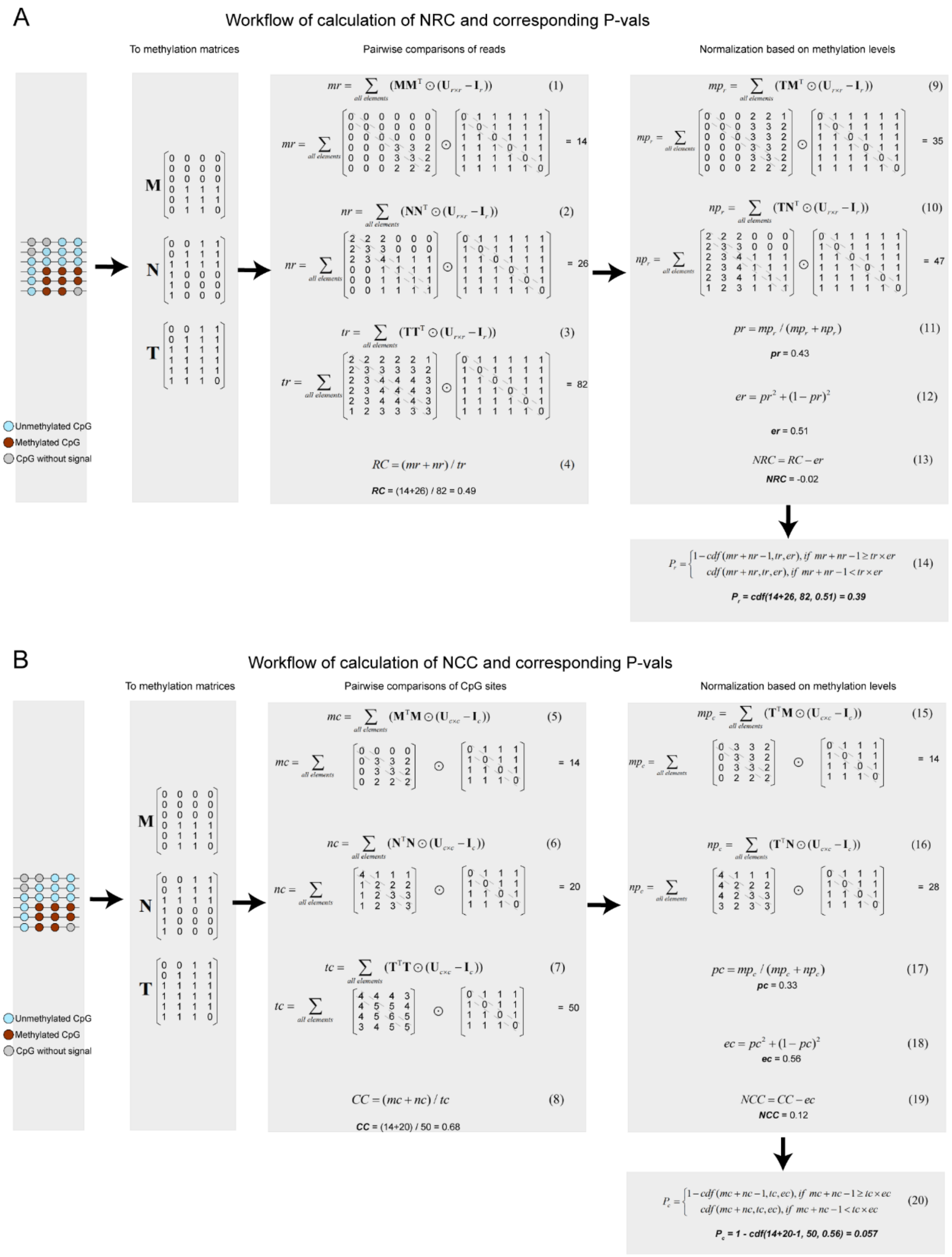


**Supplementary Fig. S2. An example used to illustrate how MeConcord works in detail.** To efficiently calculate the concordance scores across reads and CpGs, we leveraged matrix multiplication to enable accommodating thousands of reads for single loci at a fast speed and feasibly coping with missing data. Panel A and B showed the workflow of calculating NRC and NCC, respectively, and their corresponding *P*-values.

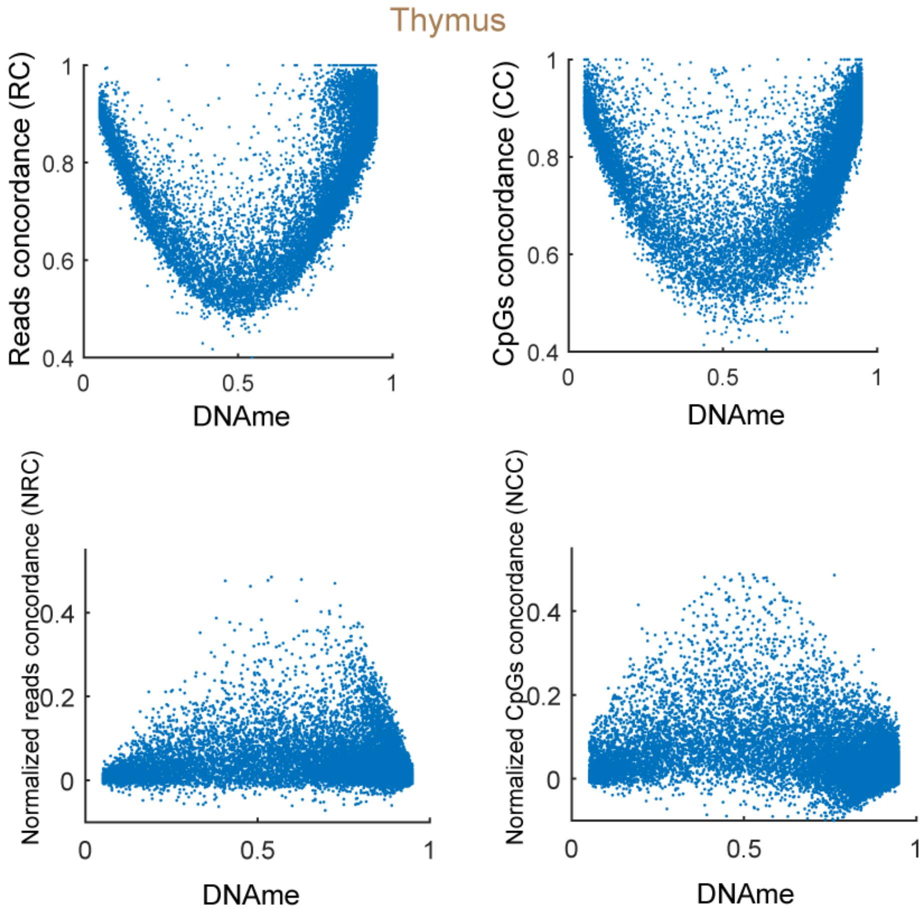


**Supplementary Fig. S3. The relationship between two metrics and DNA methylation levels in the thymus.** 150-bp bins were randomly subsampled at 10% and shown in the figure. Reads concordance and CpGs concordance are biased by DNA methylation levels, while Normalized reads concordance and normalized CpGs concordance are not biased by methylation levels.

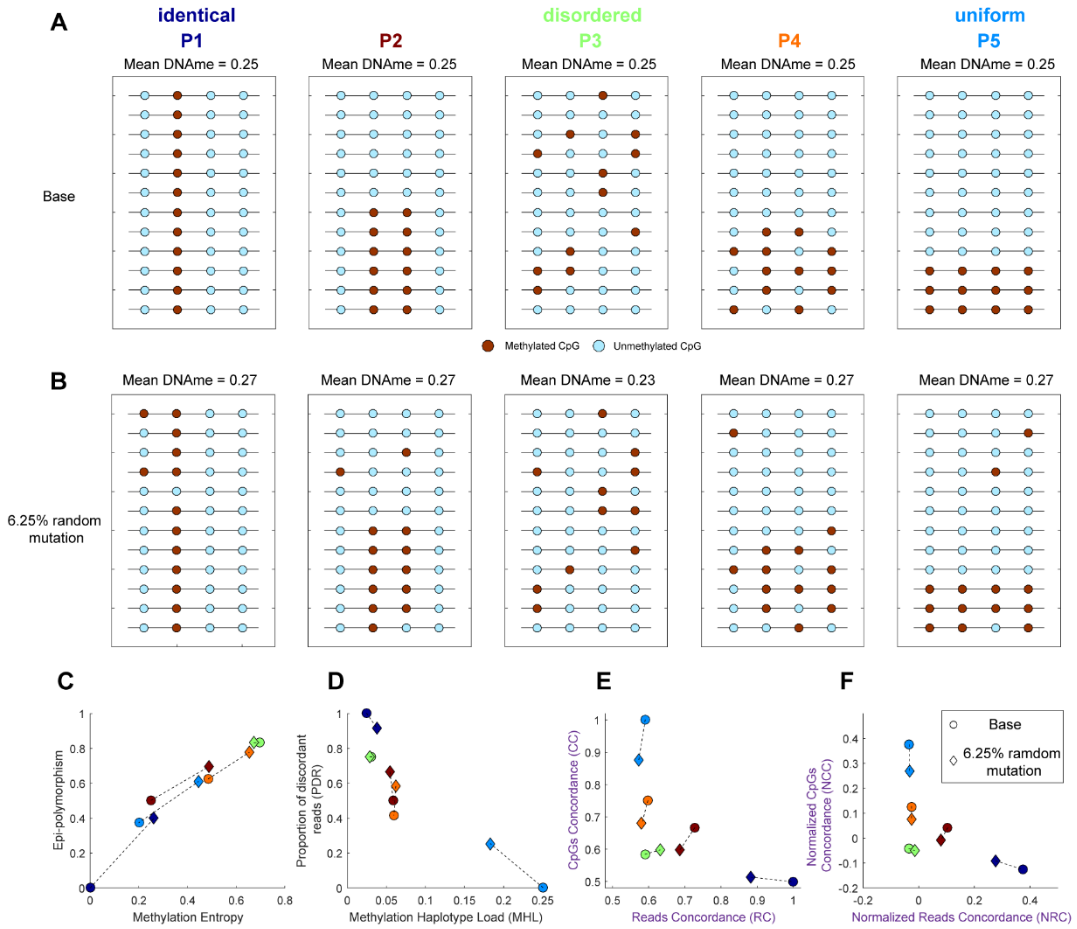


**Supplementary Fig. S4. MeConcord showed stable performance on shorter stretches** (**A-B**) Five different methylation patterns for intermediately methylated regions (methylation levels were around 0.25 for each pattern) with 4 CpG sites and 12 reads. A: methylation patterns without methylation mutations. B: methylation patterns with 6.25% (3/48) random methylation mutations to mimic real experiment data. (**C–F**) The scatter plots of metrics measuring regional methylation heterogeneity for 10 methylation patterns including both the base and mutated patterns. **C** and **D** show 4 metrics used in previous studies, exhibiting poor performance in distinguishing different methylation patterns. However, **E** and **F** show metrics used in this study, exhibiting good performance in distinguishing different methylation patterns, especially for P1, P3, and P5, and in coping with noise.

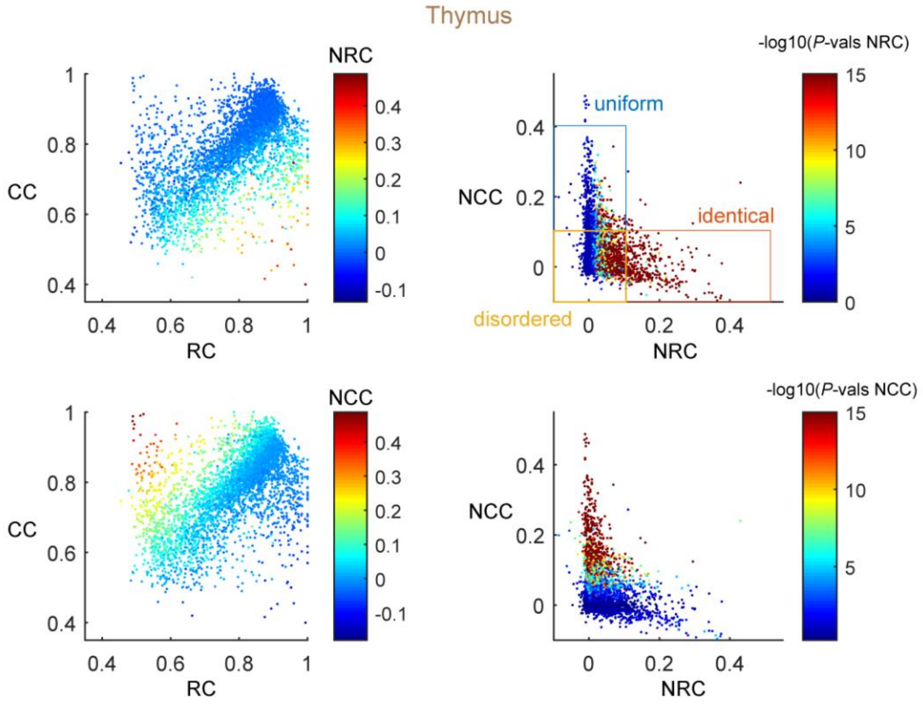


**Supplementary Fig. S5. The distribution of concordance metrics in the thymus.** Normalized reads concordance and normalized CpGs concordance showed the preferable performance to distinguish different methylation patterns.

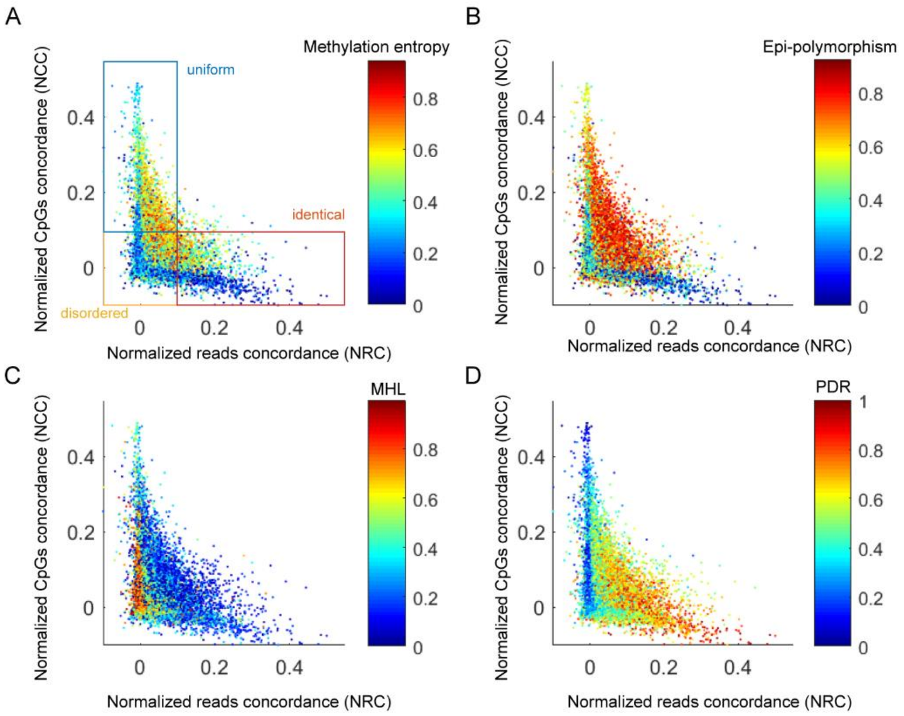


**Supplementary Fig. S6. Performance of previously published metrics in distinguishing different patterns with real data.** (**A-D**) The relationship between previously published metrics and our metrics (*NRC*, *NCC*). methylation entropy (**A**), epi-polymorphism (**B**), MHL (**C**) and PDR (**D**) were shown as colors of points. They could not efficiently distinguish three different methylation patterns. We noticed that methylation entropy and epi-polymorphism could not deal with missing data, so we used 4 CpG sites with the highest coverages in 150-bp bins to calculate two metrics.

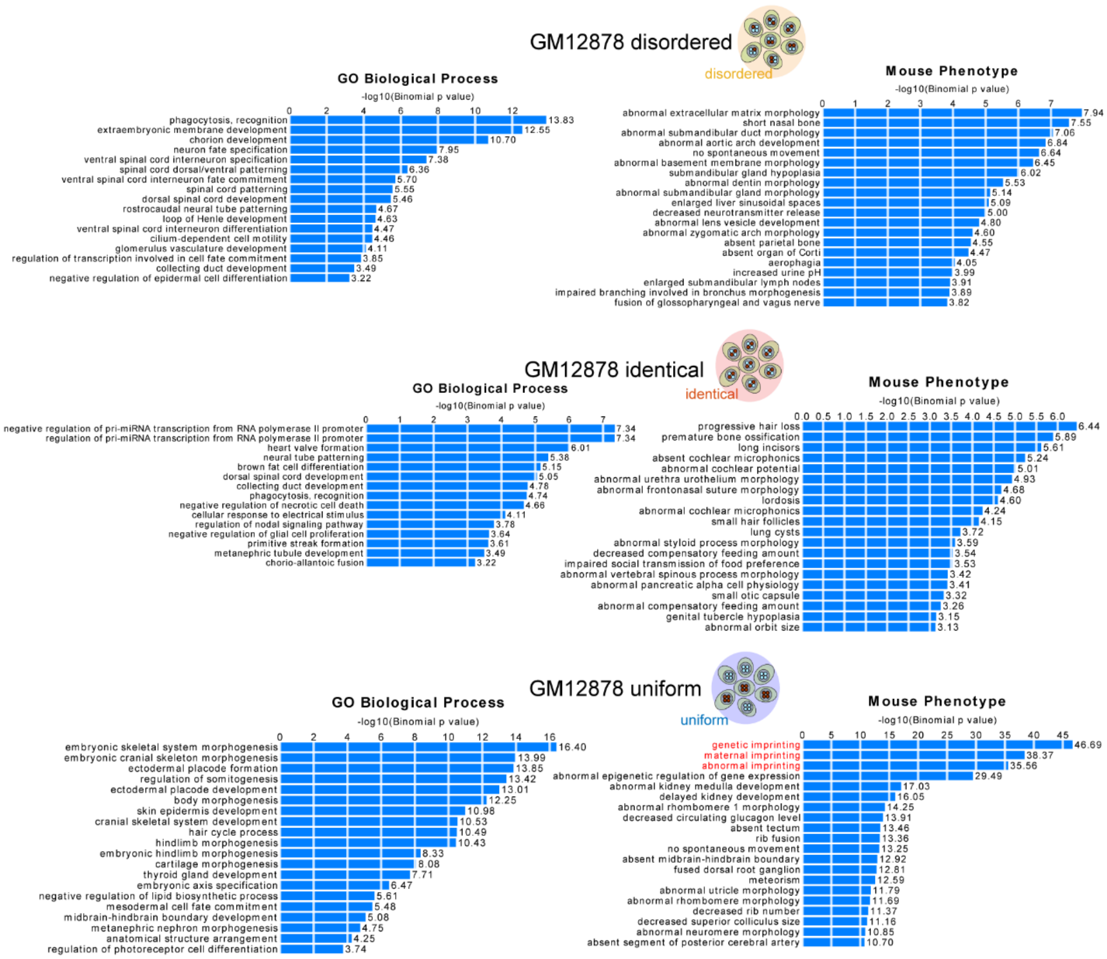


**Supplementary Fig. S7. GREAT enrichment results for GM12878 disordered, identical, and uniform bins.** GO biological process and mouse phenotype were shown. Top 5000 bins (disordered, identical, and uniform bins were sorted by reads numbers, normalized reads concordance levels, and normalized CpGs concordance levels, respectively) were selected for enrichments. Notably, uniform bins showed high enrichments for genetic imprinting, maternal imprinting, abnormal imprinting (Binomial $P$-values < $1 \times 10^{-30}$).

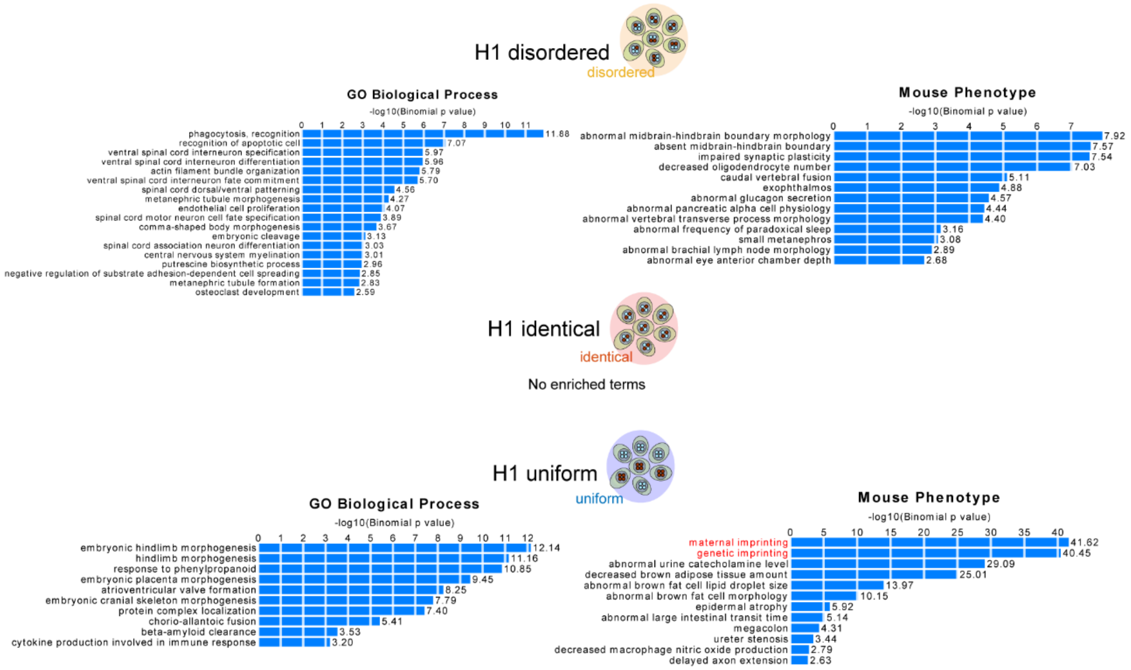


**Supplementary Fig. S8. GREAT enrichment results for H1 disordered, identical, and uniform bins.** GO biological process and mouse phenotype were shown. Top 5000 bins (disordered, identical, and uniform bins were sorted by reads numbers, normalized reads concordance levels, and normalized CpGs concordance levels, respectively) were selected for enrichments. Notably, uniform bins showed high enrichments for genetic imprinting, maternal imprinting (Binomial *P*-values < $1\times10^{-40}$). Besides, uniform bins were also enriched for embryonic morphogenesis (Binomial *P*-values < $1\times10^{-7}$).

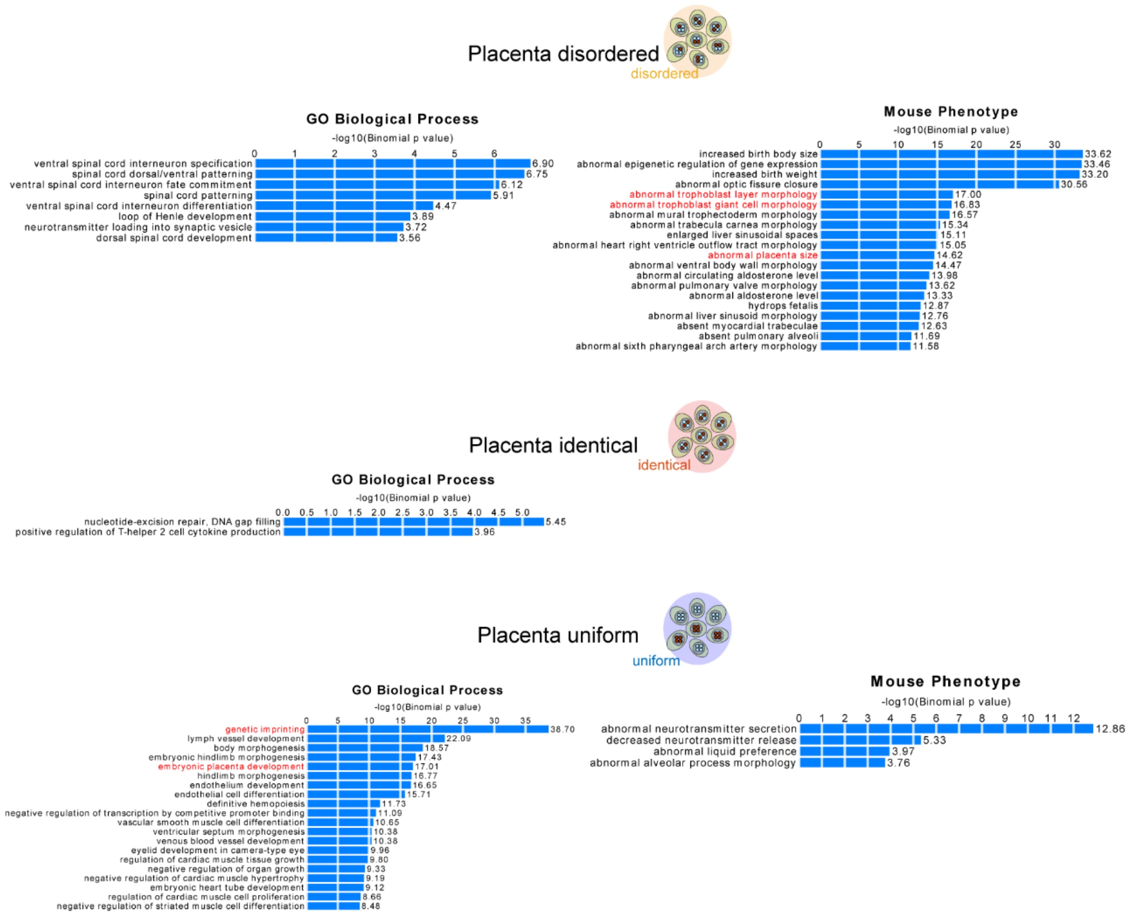


**Supplementary Fig. S9. GREAT enrichment results for placenta disordered, identical, and uniform bins.** GO biological process and mouse phenotype were shown. Top 5000 bins (disordered, identical, and uniform bins were sorted by reads numbers, normalized reads concordance levels, and normalized CpGs concordance levels, respectively) were selected for enrichments. Notably, disordered bins showed high enrichments for trophoblast (will develop into a large part of the placenta) morphology (Binomial *P*-values < $1\times10^{-10}$) and abnormal placenta size (Binomial *P*-values < $1\times10^{-10}$). Besides, uniform bins enriched for genetic imprinting (Binomial *P*-values < $1\times10^{-30}$) and embryonic placenta development (Binomial *P*-values < $1\times10^{-10}$).

**Supplementary Table S1. Metrics for Fig. 4E top example**

| | Chr13:27,975,852-27,976,001 | | | | [min, max] |
|---|---|---|---|---|---|
| | Cellular Senescence | | Tumorigenesis | | |
| Samples | P1 | S1 | Lung_normal1 | Lung tumor1 | |
| Methylation levels | 0.02 | 0.25 | 0.06 | 0.3 | [0,1] |
| Methylation entropy | 0 | 0.46 | 0.12 | 0.54 | [0,1] |
| Epi-polymorphism | 0 | 0.69 | 0.18 | 0.68 | [0,1] |
| MHL | 0 | 0.007 | 0.0002 | 0.04 | [0,1] |
| PDR | 0.1 | 0.54 | 0.24 | 0.33 | [0,1] |
| NRC | 0 | 0.08 | 0 | -0.008 | [-0.2,0.5] |
| NCC | 0 | 0.06 | 0.01 | 0.34 | [-0.2,0.5] |
| Pr (P values) | 0.46 | 5.40E-10 | 0.48 | 0.16 | [0,1] |
| Pc (P values) | 0.48 | 0.001 | 0.1 | 0 | [0,1] |

We noticed that methylation entropy and epi-polymorphism could not deal with missing data, so we used 4 CpG sites with the highest coverages in 150-bp bins to calculate two metrics.

**Supplementary Table S2. Metrics for Fig. 4E bottom example**

| | Chr12:128,853,775-128,853,924 | | | | [min, max] |
|---|---|---|---|---|---|
| | Cellular Senescence | | Tumorigenesis | | |
| Samples | P1 | S1 | Lung_normal1 | Lung tumor1 | |
| Methylation levels | 0.02 | 0.29 | 0.02 | 0.35 | [0,1] |
| Methylation entropy | 0 | 0.42 | 0.1 | 0.53 | [0,1] |
| Epi-polymorphism | 0 | 0.64 | 0.15 | 0.68 | [0,1] |
| MHL | 0 | 0.004 | 0 | 0.1 | [0,1] |
| PDR | 0.19 | 0.83 | 0.14 | 0.33 | [0,1] |
| NRC | 0 | 0.16 | 0 | -0.01 | [-0.2,0.5] |
| NCC | 0.002 | 0.01 | 0 | 0.39 | [-0.2,0.5] |
| Pr (P values) | 0.42 | 0 | 0.49 | 0.006 | [0,1] |
| Pc (P values) | 0.76 | 0.96 | 0.36 | 0 | [0,1] |

We noticed that methylation entropy and epi-polymorphism could not deal with missing data, so we used 4 CpG sites with the highest coverages in 150-bp bins to calculate two metrics.
